# On the Possibility of the Deformation of Mg and Alloys Without Preheating of Initial Billets: Understanding Their Corrosion Performance via Electrochemical Tests

**DOI:** 10.3390/ma17246182

**Published:** 2024-12-18

**Authors:** Anna Dobkowska, Jiri Kubasek

**Affiliations:** 1 Faculty of Materials Science and Engineering, Warsaw University of Technology, Woloska, 02-507 Warsaw, Poland; 2 Department of Metals and Corrosion Engineering, University of Chemistry and Technology, Technicka 5, 168 28 Prague 6, Czech Republic

**Keywords:** magnesium alloys, plastic deformation, microstructure, corrosion

## Abstract

Due to limited slip systems activated at room temperature, the plastic deformation of Mg and its alloys without any preheating of initial billets is significantly limited. To overcome those issues, new methods of severe plastic deformation are discovered and developed. One such example is extrusion with an oscillating die, called KoBo. This method, due to the oscillations of reversible die located at the end of extruded, introduces material into the plastic flow, and thus, enables deformation without preheating of the initial billets of metals that are hard to deform. Such solution is important from an industrial point of view and may lead to serious savings and reduction in carbon dioxide emission to the atmosphere. Therefore, this paper focuses on the possibility of KoBo extrusion of hcp-structured Mg alloys with different chemical compositions and includes comparison of their corrosion resistance using short-term electrochemical tests. In order to have a broad view of the problem presented, we compared the electrochemical behavior of the following groups of Mg materials: pure Mg, Mg-Al-Zn, Mg-Li, and Mg-Y-RE. It was stated that the KoBo method performed at room temperature improves the corrosion resistance of pure Mg when compared to the initial billet and the alloys which belong to the Mg-Al-Zn, Mg-Li, and Mg-Y-RE series. The presented study shows that different corrosion trends are observed for traditionally deformed alloys, and they significantly vary from nascent developments, such as KoBo extrusion. Therefore, it is crucial to widely study those methods because it may be a path leading to long-lasting solution to the formability limitations of Mg-based metallic systems.

## 1. Introduction

The most appropriate way to describe the role of grain size in the corrosion behavior of materials after deformation is to analyze pure metals to avoid the effect of second phases and their anodic or cathodic contribution to corrosion mechanisms. Pure Mg has a standard potential of −2.373 V vs. SHE, while its free corrosion potential is more positive (toward −1.6 V), indicating that a surface layer is present on the pure Mg, limiting its contact with the solution [1,2]. This corrosion layer is not fully protective, but limits corrosion reactions on the substrate of Mg metal to some extent. To date, many researchers focused on finding clear relationship between grain size and corrosion behavior for Mg alloys [3,4,5,6]. As is commonly known, grain size is a predominant factor affecting corrosion resistance, and it might be expected that a universal law describing it would exist. Unfortunately, this is impossible for plastically deformed polycrystalline materials because grain refinement itself is also related to the dislocation density and distribution, as well as the internal strain induced during deformation [7]. Another important factor is the tremendous role of an intermetallic phase, β-Mg_17_Al_12_, in mechanical and corrosion properties, which has already been described [8,9,10]. When AZ61 (6 wt.% Al, 1 wt.% Zn) was subjected to ECAP, precipitation strengthening was observed [11]. The large proportion of β-Mg_17_Al_12_ hindered dislocation movement, leading to an increased dislocation density, thus causing work hardening. Nevertheless, such a large amount of this type of intermetallic phase may promote corrosion reactions by forming microgalvanic reactions with the Mg matrix [12]. From the opposite point of view, β-Mg_17_Al_12_ may form a barrier against spreading corrosion reactions [13,14,15]. Therefore, the dual role of β-Mg_17_Al_12_ in the corrosion resistance of AZ-type alloys needs to be further examined. When it comes to dual-phase structured Mg-Li alloys, two crucial factors dominating their corrosion performance have been recently described. Zeng et al. [16] suggested that Li leads to the formation of a thin film, which may play a protective role on the surface. It was also proposed that corrosion of the dual-phase structured AZ31+7.5Li traditionally extruded at 350 °C is a result of microgalvanic interaction between the α(Mg) and β(Li) phases and their relative distribution [17]. The more equal the fractions of α(Mg) and β(Li), the less intense were the resulting corrosion reactions. Besides the mentioned factors, texture intensity also affects the corrosion behavior especially in alloys containing Y and RE as alloying elements.

All of the above mentioned factors are typical for Mg alloys plastically deformed at high temperature. Lastly, our research groups proved that Mg and its alloys may be formed without preheating of the initial billet which is very unusual for hcp-structured metals [18]. The method used for the plastic deformation of Mg alloys at room temperature is a modified extrusion with an oscillating die located at the end of extruded (as described in [19], called KoBo. Since Mg and its alloys are characterized by poor corrosion characteristics, which limit their widespread use in industrial applications, it is of crucial importance to carry out a systematic study about the factors affecting their corrosion after extrusion using nascent developments, such as KoBo, which may lead to many economic benefits in industrial applications (elimination of furnaces from production routes, reduction in gas emission, faster deformation processes, etc.). Therefore, the main aim of this paper is to select and describe the predominant factors influencing various groups of KoBo-deformed Mg alloys and to specify the most corrosion-resistant group of Mg alloys after KoBo extrusion based on fast electrochemical corrosion tests.

## 2. Materials and Methods

The electrochemical behavior indicating corrosion resistance of various Mg alloys processed using extrusion with an oscillating die (KoBo) without preheating of the initial billet is investigated and compared. The materials for this research were selected based on our previous studies, where the corrosion resistance of pure Mg [20], AZ31 [21], AZ61 [22], AZ31+4Li [23], AZ31+7.5Li [24], and WE43 [25] extruded using KoBo at various extrusion ratios has been described. For this research, the alloys with the highest corrosion resistance analyzed in the previous studies have been chosen, and they are specified in Table 1.

The electrochemical measurements were performed in naturally aerated 0.01 M NaCl solution using a Gamry FAS1 600+ potentiostat equipped with three electrodes: platinum as the counter electrode, Ag/AgCl as the reference electrode, and the measured sample as the working electrode. The electrolyte was made up using analytical-grade reagents and distilled water. The samples were immersed for 1 h, and electrochemical impedance spectroscopy was performed over a frequency range of 0.01 Hz–10,000 Hz. Immediately afterward, potentiodynamic polarization tests were carried out over a range from 0.2 V below E_OCP_ to 1.0 V vs. Ref (a scan rate of 5 mV/s was used). At least three tests were performed for each alloy. The EIS and polarization curves were fitted using the Gamry Echem software version 5.58. Corrosion damage was characterized after 1 h of immersion in 0.1 M NaCl. Before electrochemical tests, samples were polished using water-free diamond suspensions (3 and 1 µm). The morphology of corrosion damage was observed, after chemical removal of the corrosion products in aqueous CrO_3_ solution (4 min, 180 g/L CrO_3_), using a scanning electron microscope (SEM, Hitachi SU8000, Japan). Additionally, the corrosion rate of the materials was calculated using the hydrogen release method [26,27,28,29]. To ensure the reproducibility of the results, at least three specimens from each composition were tested.

## 3. Results

**Figure 1:**
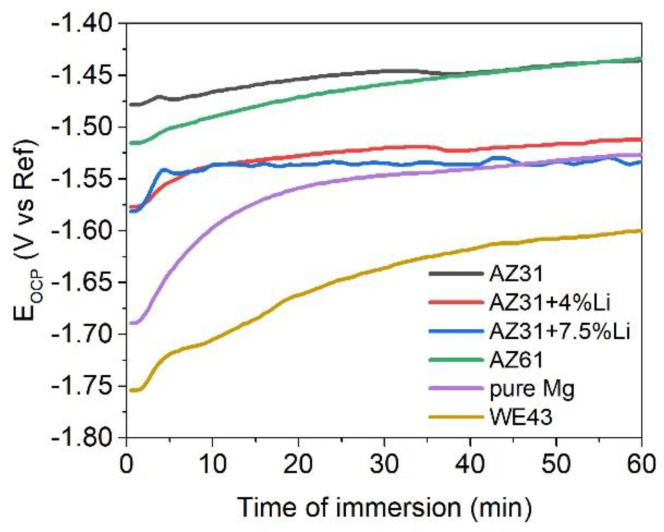
Open circuit potential (E_OCP_) evolution during 1 h of immersion in naturally aerated 0.1 M NaCl.

**Figure 2:**
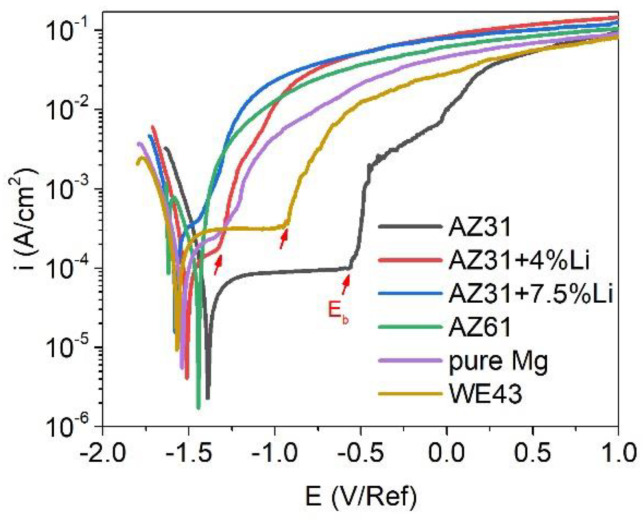
Potentiodynamic polarization curves recorded after 1 h of immersion in naturally aerated 0.1 M NaCl.

The evaluation of the open circuit potential (E_OCP_) of the investigated materials during 1 h of immersion in 0.1 M NaCl is presented in Figure 1. The highest values of the E_OCP_ were observed for AZ31 and AZ61, and their average values were −1.45 V/Ref and −1.46 V/Ref, respectively. The 4 and 7.5 wt.% of Li addition shifted the E_OCP_ toward negative values: −1.53 V/Ref and −1.54 V/Ref, respectively. The lowest values of E_OCP_ were observed for pure Mg and WE43: −1.56 V/Ref and −1.65 V/Ref, respectively. Inspection of the anodic branch of the polarization curves in Figure 2 determined the active dissolution of the AZ61, while the rest of the alloys underwent localized corrosion. A characteristic plateau was observed on the anodic branch of AZ31, WE43, pure Mg, AZ31+4Li, and AZ31+7.5Li, and a typical inflection point indicating the breakdown potential, E_b_, was also noted (labeled by the red arrows in Figure 2). The difference between E_b_ and E_corr_ describes the resistivity to pitting corrosion, ∆E [30,31]; the broader the difference, the higher the resistance to pitting. The numerical values of the resistivity to pitting are shown in Table 2. Undoubtedly, the highest pitting susceptibilities were in the cases of pure Mg and the alloys with Li addition. The most stable corrosion layers were formed on the AZ31 and WE43.

**Figure 3:**
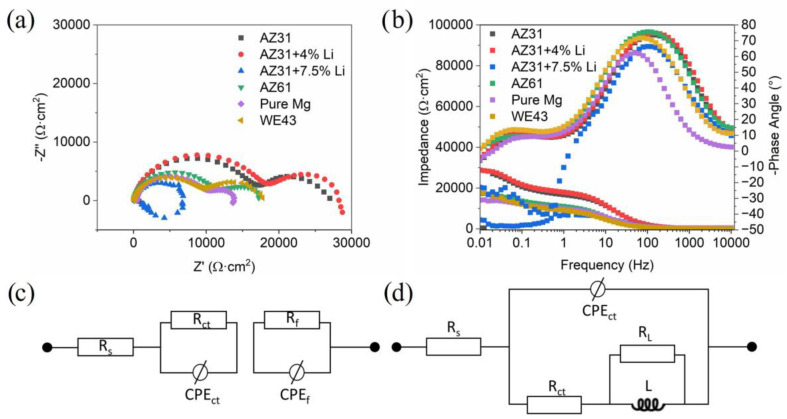
Electrochemical impedance spectroscopy (EIS) results recorded after 1 h of immersion in naturally aerated 0.1 M NaCl and presented in the form of (**a**) Nyquist plots, (**b**) Bode plots, (**c**) equivalent electrical circuit used for pure Mg, AZ31, AZ61, AZ31+4Li, and WE43 fitting, and (**d**) equivalent electrical circuit used for AZ31+7.5Li fitting.

**Figure 4:**
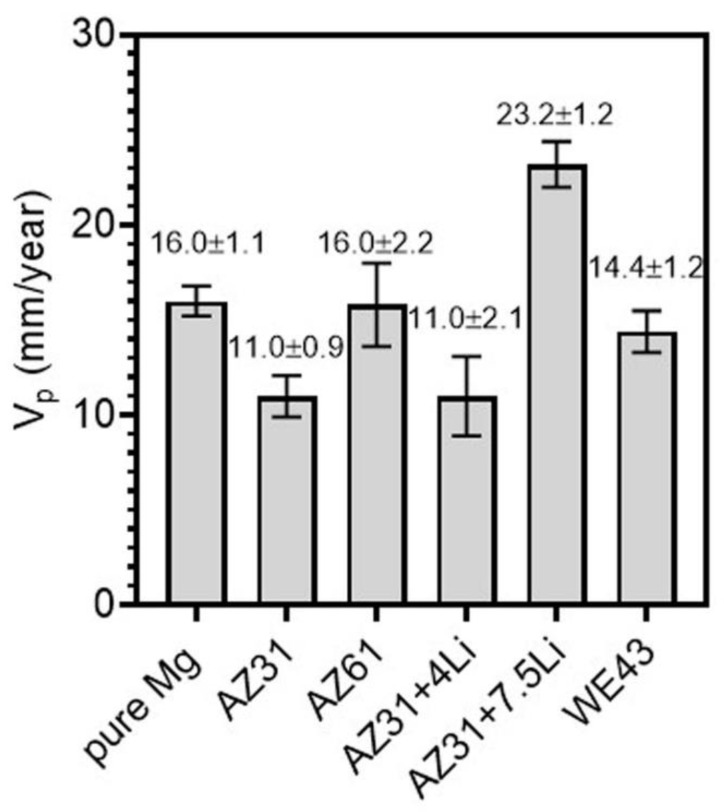
Corrosion rate calculated based on the hydrogen release method.

The Nyquist plots recorded for the investigated materials after 1 h of immersion in naturally aerated 0.1 M NaCl are shown in Figure 3a, while corresponding Bode plots are depicted in Figure 3b. All samples exhibited two semicircles, corresponding to two time constants, with the exception of AZ31+7.5Li, where the small radius of the Nyquist plot and intense inductive loop may indicate strong localized corrosion and/or precipitation of corrosion products on the surface. The Nyquist plots for pure Mg, AZ31, AZ61, AZ31+4Li, and WE43 were composed of two semicircles registered at a high and medium frequency range. The first one was related to the charge transfer resistance at the double layer interface, while the second one corresponded to the corrosion products’ layer [32,33]. The equivalent electrical circuits given in Figure 3c,d were used to fit data for these samples. Here, R_S_ is the solution resistance; R_ct_ is the charge transfer resistance, and the corresponding capacitance is defined by the constant phase element (CPE_ct_). The R_f_ and CPE_f_ variables stand for the resistance of the corrosion products’ layer. In the given equivalent electrical circuits, CPE is used to compensate for the nonuniformity of the corrosion system [34]. The results from the fitting are depicted in Table 2. The equivalent electrical circuits given in Figure 3d were used to fit the data recorded for AZ31+7.5Li. They were composed of a capacitive loop described by R_ct_ and CPE_ct_ and an inductive loop defined by R_L_ and L, with the latter loop being most probably related to the initial stage of localized corrosion [35,36], although it can also be related to the precipitation of corrosion products. The behavior of the analyzed samples, with the exception of Mg-7.5Li, was very similar to each other in the analyzed medium and exhibited the presence of a protective film. The complex nature of the fitted data was supported by the corrosion rate calculated based on hydrogen release tests, Figure 4. Both the EIS results and estimated corrosion rates showed a similar tendency with respect to the corrosion behavior of the investigated materials. The least resistant was dual-structured AZ31+7.5Li, where strong dissolution of the alloy was observed. Its corrosion rate was found to be 23.2 ± 1.2 mm/year. The rest of the materials were less active in the analyzed solution. The most resistant were AZ31 and AZ31+4Li, with corrosion rates of 11.0 ± 0.9 mm/year and 11.0 ± 2.1 mm/year, respectively. The higher standard deviation out of these two samples was observed for the alloy containing Li. These alloys exhibited the highest values of R_CT_ and R_f_, suggesting that the most stable corrosion product layers are formed on their surfaces (Table 3). Less resistant than AZ31 and AZ31+4Li were AZ61, WE43, and pure Mg, and their corrosion rates were comparable to each other: 16.0 ± 2.2, 14.4 ± 1.2, and 16.0 ± 1.1 mm/year, respectively.

**Figure 5:**
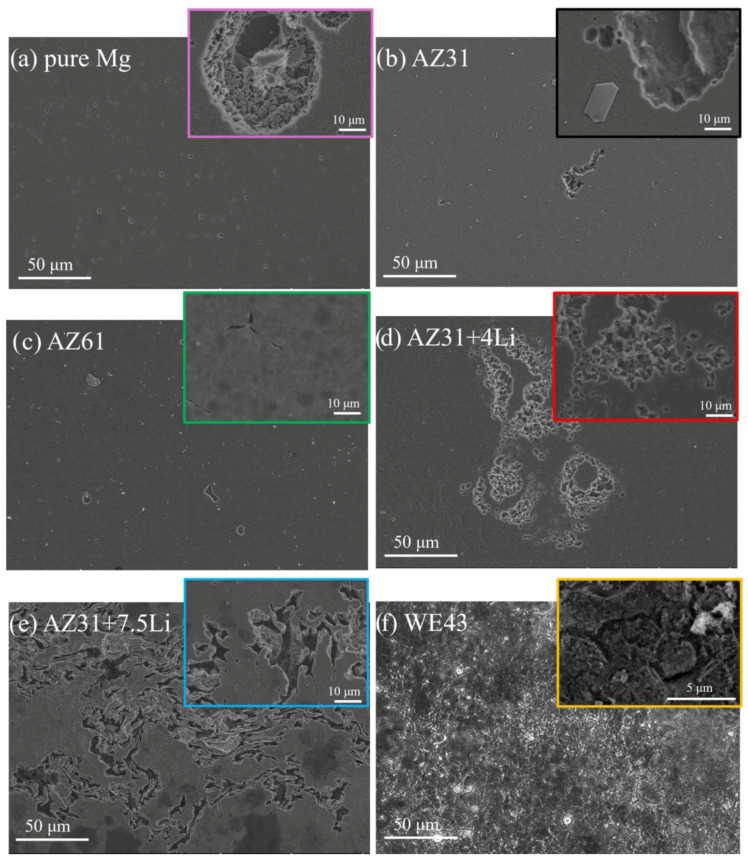
Corrosion damage characterization after chemical removal of corrosion products after 1 h of immersion in 0.1 M NaCl: (**a**) pure Mg, (**b**) AZ31, (**c**) AZ61, (**d**) AZ31+4Li, (**e**) AZ31+7.5Li, and (**f**) WE43.

Figure 5a–f show the surface morphologies of the corroded samples after removing the corrosion products. Many small and round pits were observed on the surface of pure Mg (Figure 5a); in random locations, they propagated into the depth of the material (inset in Figure 5a). In the case of AZ31 (Figure 5b) and AZ61 (Figure 5c), besides small round pits, larger and deeper pitting holes interlinked with one another on the surface were observed. The pits propagated on the surface of AZ31 are as deep as those formed on pure Mg (inset in Figure 5b). As shown in the inset in Figure 5c, corrosion damage in triple points was formed on the AZ61. The surface of the AZ31 with Li was more damaged; however, the corrosion damage differed depending on the amount of Li present. On the surface of the single-α(Mg)-phase AZ31+4Li, corrosion in the form of threads proceeding along the grain boundaries was observed (Figure 5d). The dual-phase structured alloy exhibited a different mode of degradation related to microgalvanic corrosion between the α(Mg) and β(Li) phases (Figure 5e). This phenomenon is explained in detail in our previous study [24]. In the case of WE43, the corrosion mechanism propagated along the grain boundaries (Figure 5f). The corroded grain boundaries are depicted at higher magnification in the inset in Figure 5f.

## 4. Discussion

The extrusion with an oscillating die (KoBo) method exhibits great potential for Mg alloy deformation without preheating of the initial billet. It may significantly reduce the grain size and redistribute secondary phases in the microstructure. It is noteworthy that the grain refinement does not depend on the extrusion ratio, but on the intensity of dynamic recrystallization (DRX) triggered by the oscillations of the reversible die. As a result of this phenomenon, the corrosion resistance of Mg alloys changes in a nonlinear manner. Of course, the corrosion resistance of KoBo-extruded alloys is dependent on the alloy chemistry, but based on the data published so far, the predominant role seems to be played by grain size and dislocation distribution.

Based on the presented results of electrochemical behavior and hydrogen release, the corrosion resistance of the analyzed materials may be ordered as follows: AZ31 > AZ31+4Li >> WE43 > pure Mg > AZ61 >>> AZ31+7.5Li. The order of the corrosion resistance of each material were analyzed based on the detailed description of the microstructure presented in our previous works, and the results are shortly summarized in Table 4.

As per results of this work, lower corrosion rate estimated via electrochemical measurements was observed for α(Mg) alloys than for dual-structured α(Mg)+β(Li) alloys. The most resistant in the analyzed medium was AZ31. As shown in [21], it has a grain size of 4.38 µm and a preferential orientation of the grain toward (10$\bar{1}0$) and (2110). The Al fully dissolved in the solution, and no β-Mg_17_Al_12_ was formed. Similar level of corrosion resistance was observed for the AZ31 alloyed with 4 wt.% of Li. In this case, nanosized β-Mg_17_Al_12_ distributed in the grain interiors was observed, creating a barrier against spreading corrosion reactions caused by the Li in the alloy [23]. Lower corrosion resistance and also corrosion products being formed on the surface in some kind of “protective” layer, was characteristic for WE43, pure Mg, and AZ61. The presence of nanosized β-Mg_17_Al_12_ at the grain boundaries promotes microgalvanic corrosion in AZ61, thus lowering its corrosion resistance when compared to AZ31 [22]. Surprisingly, pure Mg had a higher corrosion rate than AZ31. Since the grain sizes for pure Mg and AZ31 were similar, the factor favoring higher corrosion resistance lay in the crystallographic orientation of the grains formed in AZ31. In this case, besides grains oriented to (10$\bar{1}0$) and (2110), grains with a (0001) orientation were present. In this relation, the grains with an (0001) orientation parallel to the corroded surface are more corrosion resistant than grains with orientations (10$\bar{1}0$) and (2110) [37].

During KoBo, materials enters to the state of plastic flow, therefore the reversibly oscillating of the die at the end of the extruded enables deformation of Mg alloys. Simultaneously, the presence of the complex strain conditions formed as a result of the die oscillations resulted in the accumulation of high density of defects in the microstructure of the extruded material. This in turn led to the pile up of dislocations in the refined grains. As stated in [21], in the KoBo extruded AZ31 alloy, the main factors affecting corrosion resistance were dislocation density and its distribution. Consequently, despite the KoBo extrusion reducing grain size from a coarse to a micrometer scale, it did not improve the corrosion resistance of the alloy. A similar situation was observed for the AZ61 [22,38]; however, strong microgalvanic reactions between β-Mg_17_Al_12_ and the Mg matrix occurred, overwhelming the role of other microstructural components in its corrosion resistance [39]. WE43 has a completely different chemical composition than that of the other Mg alloys, and here, the predominant role in the corrosion mechanism may be caused by the presence of tiny precipitates located in this alloy [25], which has a corrosion resistance similar to AZ61 and pure Mg. Nevertheless, the role of small second phases must be further analyzed.

## 5. Conclusions

Based on the performed tests and reviewed data, the following may be concluded:

- KoBo extrusion enables the deformation of Mg and its alloys without preheating of the initial billet. The obtained materials are without cracking and reduce the diameter of the ingots significantly, resulting in the production of long elements, such as rods or wires. The next step of the KoBo improvement should be research on the possibility of thin-walled structure fabrication;
- KoBo extrusion improves the corrosion resistance of the initial ingots of Mg and its alloys; however, there is no clear relationship between grain refinement and corrosion resistance improvement, and smaller grain size does not always lead to higher corrosion resistance. The properties of the specific alloy must be treated as a separate case, since the intensity of the dynamic recrystallization is related to the extrusion ratio and strain induced by the reversible die;
- The corrosion resistance of the KoBo-extruded alloys depends mainly on the alloy chemistry and its microstructure; however, it is also dominated by the formation of stresses during extrusion with an oscillating die. Importantly, we do not have any clear information about the specific temperature inside the extruder. The effect of the internal heating resulting from the friction of the material and the extruder during processing should be further investigated;
- In the current research on magnesium alloys, the imperative issue of balancing the structural characteristics and mechanical properties of the alloys remains to be resolved. A reduction in the deformation temperature of Mg alloys will generate savings and reduce CO_2_ gas emissions, which is crucial to ensure the sustainable growth of industry.

## Figures and Tables

**Table 1:** Selection of Mg materials in order to make a comparative analysis of their corrosion resistance.

Materials	Pure Mg	AZ31	AZ61	AZ31+4Li	AZ31+7.5Li	WE43
Extrusion ratio	7:1	10:1	7:1	10:1	10:1	5:1

**Table 2:** The characteristic parameters calculated based on the extrapolation of linear polarization (E_corr_—corrosion potential, E_b_—breakdown potential).

Parameters	Pure Mg	AZ31	AZ61	AZ31+4Li	AZ31+7.5Li	WE43
E_corr_ (V vs. Ref)	−1.54	−1.39	−1.44	−1.51	−1.58	−1.57
E_b_ (V vs. Ref)	−1.34	−0.56	-	−1.33	−1.45	−0.94
∆E	0.20	0.83	-	0.18	0.13	0.63

**Table 3:** Parameters fitted from EIS results using data shown in Figure 3.

Materials	R_s_ (Ω∙cm^2^)	R_ct_ (Ω∙cm^2^)	CPE_ct_ (µSs^a^/cm^2^)	a_1_	R_L_ (Ω∙cm^2^)	L (H∙cm^2^)	R_f_ (Ω∙cm^2^)	CPE_f_ (µSs^a^/cm^2^)	a_2_
Pure Mg	61	1242	0.000018	0.94	N/A	N/A	634	0.000840	0.84
AZ31	22	2266	0.000008	0.85	N/A	N/A	1420	0.000120	0.86
AZ61	10	1454	0.000019	0.94	N/A	N/A	1016	0.001688	072
AZ31+4Li	17	2357	0.000009	0.93	N/A	N/A	1603	0.000155	0.71
AZ31+7.5Li	17	183	0.000025	0.88	691	182	N/A	N/A	N/A
WE43	14	1201	0.000027	0.92	N/A	N/A	1294	0.001756	0.76

**Table 4:** Summary of the basic microstructural features characteristic for the investigated materials.

Material	Phase Structure	Grain Size (µm)	Second Phases	Grain Orientation	Ref.
**Pure Mg**	α(Mg) phase	3.88 µm; uniformly distributed	N/A	(10$\bar{1}0)$ and (2110)	[21]
**AZ31**	α(Mg) phase	4.38 µm; uniformly distributed	Coarse Al_5_Mn_8_	(10$\bar{1}0$) and (2110) with some randomly present (0001)	[21]
**AZ61**	α(Mg) phase	6.6 µm uniformly distributed	Coarse Al_5_Mn_8_ and nano β-Mg_17_Al_12_ located at grain boundaries	(10$\bar{1}0$) and (2110)	[22]
**AZ31+4Li**	α(Mg) phase	3.24 µm; uniformly distributed	Coarse AlLi and β-Mg_17_Al_12_ (nano) distributed in the grain interiors	(10$\bar{1}0$) and (2110)	[23]
**AZ31+7.5Li**	α(Mg)+β(Li)	No EBSD data, based on SEM a few µm	Coarse AlLi and MgLi_2_Al with size around 1 µm, β-Mg_17_Al_12_ (nano) distributed in the grain interiors	No EBSD data	[24]
**WE43**	α(Mg) phase	3.3 µm; uniformly distributed	Mg_41_Nd_5_, Mg_24_Y_5_, Mg_12_Nd, and nano ternary Mg-Nd-Y	Random	[25]

## Data Availability

The original contributions presented in this study are included in the article. Further inquiries can be directed to the corresponding authors.
